# Use of tunneled pleural catheters in chronic empyema: Two case reports and brief review of the literature

**DOI:** 10.1016/j.rmcr.2022.101754

**Published:** 2022-10-11

**Authors:** Ilana Roberts Krumm, Yaron B. Gesthalter

**Affiliations:** Department of Medicine, Division of Pulmonary, Critical Care, Allergy, and Sleep Medicine, University of California San Francisco, 513 Parnassus Ave, Box 0111, San Francisco, CA, 94143, USA

**Keywords:** Empyema, Tunneled pleural catheter, Pleuroscopy, Pleural infection, TPC, tunneled pleural catheter, VATS, video-assisted thorascopic surgery

## Abstract

The incidence of empyema is increasing worldwide, which, coupled with the aging global population, makes the non-surgical management of pleural space infections increasingly important. Despite this, there remains no consensus for management of chronic empyema in those patients who are not surgical candidates and do not get adequate source control with chest tube and intra-pleural lytic therapy, particularly for patients with non-expandable lungs. We reviewed the literature regarding non-surgical management of chronic empyema and present two cases that support the use of pleuroscopy in conjunction with tunneled pleural catheters for management of chronic empyema in non-surgical candidates.

## 1 Background

Pleural space infections are associated with high morbidity and mortality, contribute substantial cost to our healthcare system, and are increasing in incidence across Europe and North America [1,2]. A review of admissions for pleural infections to English hospitals between 2008 and 2018 showed increasing incidence in empyema without improvement in patient outcomes [3] and similar increases have been shown in North America [4,5]. The most marked increase in empyema rates occurred in populations aged ≥60 years, with an increase of 194% over a decade [3]. This, coupled with our aging global population, makes management of empyema a pressing issue, particularly in cases not suitable for surgical intervention, which is considered the gold standard of therapy [6]. Current evidence supports the use of intrapleural lytic therapy, which has been shown to decrease need for surgical intervention [7]. However, there is limited guidance for management of patients who are not surgical candidates and do not get adequate source control with chest tube drainage and interpleural lytic therapy. We report two cases of empyema in patients, both of whom were not surgical candidates, managed with medical pleuroscopy and simultaneous tunneled pleural catheter (TPC) placement, and summarize the literature regarding non-surgical management of chronic pleural infection.

## 2 Case #1

A 73-year-old woman with history of diverticulitis resulting in colovaginal fistula and requiring colectomy, with a post-operative course that was complicated by an intra-abdominal abscess and a left sided Proteus mirabilis empyema that occurred two months after her initial colectomy. She underwent video-assisted thorascopic surgical (VATS) decortication and completed a 28-day course of piperacillin-tazobactam. The patient had months of progressive dyspnea despite therapy. She had daily productive cough of thick yellow sputum, pleuritic ipsilateral chest pain and subjective chills. She had progressive decline in her functional status, 50-pound weight loss, and general weakness ultimately leading to an inability to perform activities of daily living independently.

**Fig. 1:**
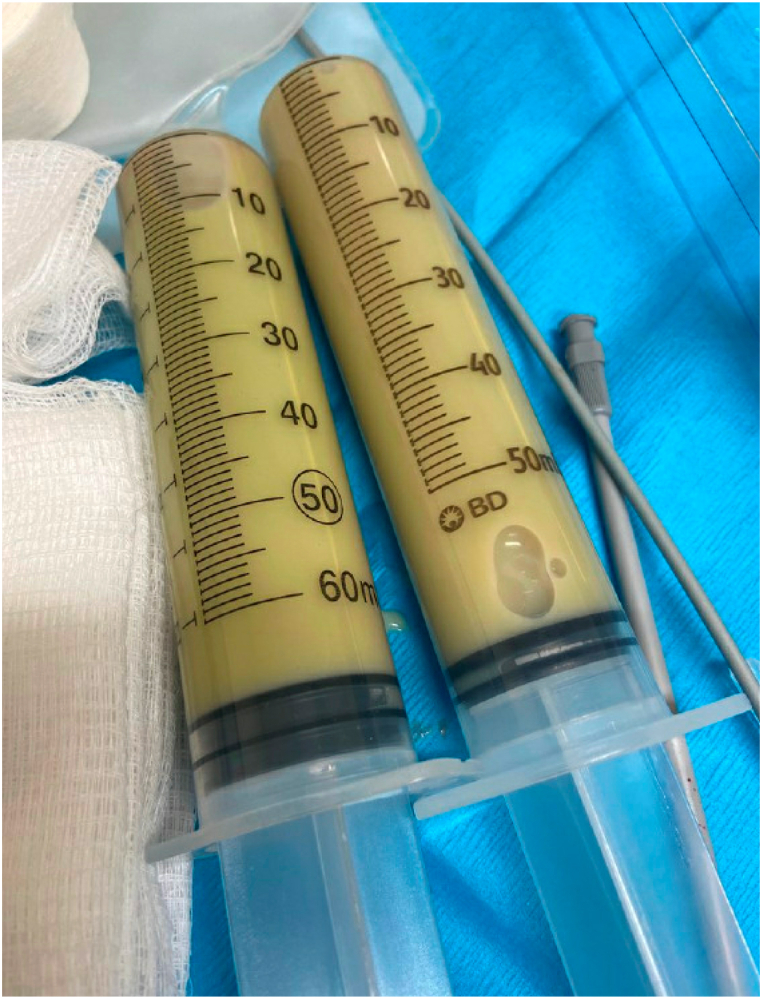
Purulent pleural fluid drained on day of presentation to pleural clinic.

In the setting of her severe deconditioning, she had a ground level fall six months after her initial decortication. When she presented to the emergency department, a CT chest demonstrated a persistent large left-sided loculated effusion. A CT guided thoracentesis was completed, aspirated fluid was notable for 30,000 WBC on cell count, suggestive of an empyema. No other studies were sent. The patient was referred urgently to our pleural clinic for further management. Upon evaluation, she was noted to be dyspneic and weak. A left-sided thoracentesis was started but converted to a 14 Fr pigtail catheter placement due to aspiration of malodorous frank pus (Fig. 1).

In total, 600mL of purulent fluid was immediately drained and sent for analysis. Pleural fluid analysis was consistent with an empyema, having a pH 6.35, glucose 9, LDH >10,000, 71 WBCs [85% neutrophils], total protein 2.6. A RAPID score at the time of presentation was calculated to be three (serum albumin 1.8, BUN 9, age >70, community acquired infection with purulent fluid), which placed the patient in the medium risk category, with an estimated 17.8% mortality at three months [8].

**Fig. 2:**
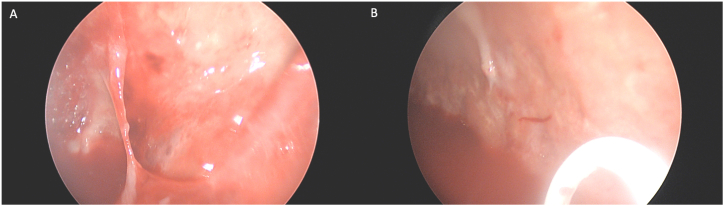
A. Pleural adhesion seen on pleuroscopy. **B.** Pleura after adhesion takedown.

She was then admitted for IV antibiotics and chest tube management. CT surgery was consulted upon admission; however, she was deemed too high of a surgical risk for repeat surgical decortication due to her poor nutritional status (Albumin 1.8). Cultures of the aspirated pleural fluid grew Proteus mirabilis while a smear that was sent noted gram-positive clusters suggesting a polymicrobial infection. In addition to IV antibiotics, she received four doses of intrapleural tPA/DNase. Despite lytic therapy, the loculated effusion persisted on imaging. The patient then underwent medical pleuroscopy with moderate sedation for a saline washout (Fig. 2), adhesion takedown and placement of tunneled pleural catheter (Pleurx, BD) 12 days after admission.

**Fig. 3:**
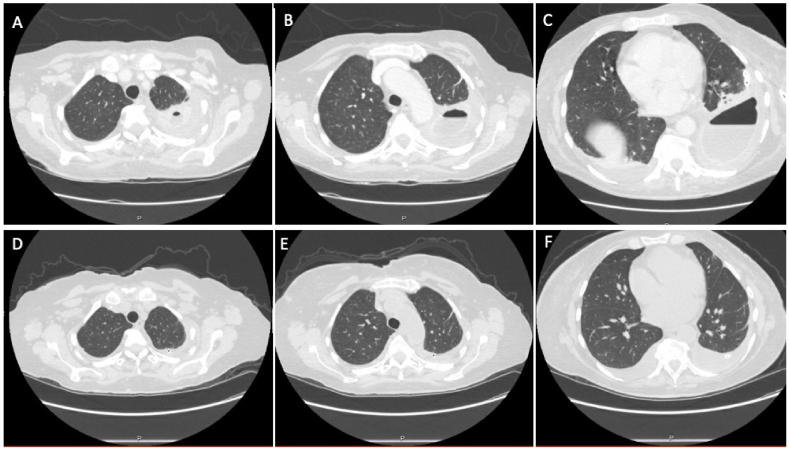
A-C Initial chest CT scan upon referral to our clinic, demonstrating intrapleural fluid collection with air-fluid level and pleural thickening. **D-F** Chest CT scan four-months after TPC placement demonstrating dramatic reduction in pleural collection.

She was discharged home three days later with home health for IV antibiotics and TPC drainage three times per week. Four months following placement of the TPC she had no residual symptoms, her inflammatory markers (CRP, WBC) nadired, repeat CT chest showed dramatic improvement of her effusion with symphysis of her pleura (Fig. 3).

**Fig. 4:**
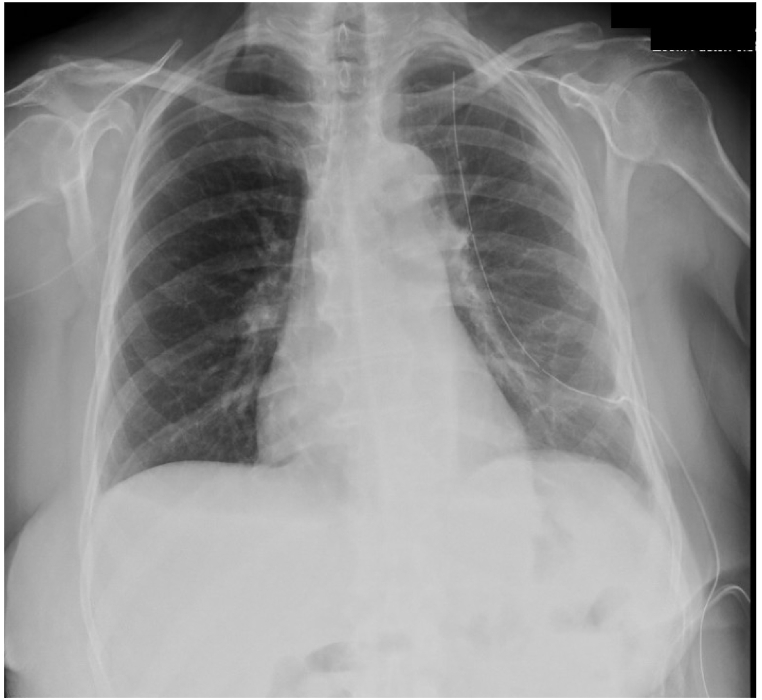
Chest radiograph just prior to TPC removal, five-months after initial placement, demonstrating small residual effusion.

Five months after placement, there was no longer output draining from the TPC. Chest radiograph demonstrated trace pleural effusion (Fig. 4) and the TPC was subsequently removed. At time of submission, there has been no interval imaging following TPC removal, however, she reports no shortness of breath, no pleuritic chest pain, and is now walking ½ a mile daily without limitation.

## 3 Case #2

A 65-year-old man with history of atrial fibrillation, non-ischemic cardiomyopathy, HCV, cirrhosis, COPD, distant pericardial and left pleural TB status post pericardial stripping 10 years prior with a residual pleural rind and complex left pleural space, with persistent loculations seen on chest imaging, was undergoing serial thoracenteses every three months, for dyspnea in the setting of recurrent pleural effusion. He was then referred to our pleural clinic for increasing frequency of left sided thoracentesis and an associated increase in dyspnea on exertion.

**Fig. 5:**
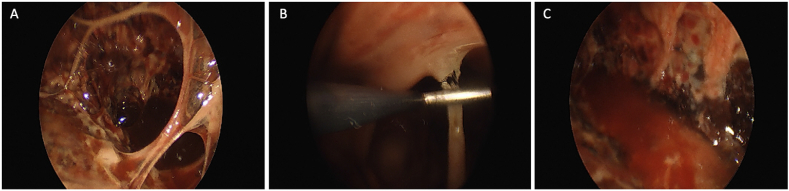
A. Pleuroscopy demonstrating significant pleural adhesions. **B**. Take-down of pleural adhesions. **C**. Pleural space after removal of adhesions.

On initial evaluation, his pleural studies were notable for an empyema (LDH >2700, Glucose<10, pH 6.89) and repeat cultures ultimately grew Staph epidermidus. He was admitted for chest tube placement, which was done under CT guidance and IV antibiotics. The chest tube was subsequently removed 10 days after insertion. No tPA was instilled given ongoing anticoagulation, mild hemoptysis, and the serosanguinous nature of his pleural fluid. One month later, he had persistent symptoms and recurrence of his pleural effusion, so a new pigtail catheter was placed on admission. Pleural fluid studies at that time demonstrated LDH >2,700, glucose<10, pH 7.12, adenosine deaminase 154, WBCs 19,000. He underwent serial irrigation with 250 cc warmed saline three times at the bedside via pigtail. VATS decortication was initially planned; however, the procedure was subsequently deferred because the patient developed flash pulmonary edema. Eleven days after pigtail placement, three doses of tPA were instilled. He was discharged 25 days after pigtail placement with a pneumostat in place. Three weeks after discharge, he followed up in pleural clinic and the pigtail catheter was removed. At one-month follow-up the patient had persistent dyspnea, with interval re-accumulation of pleural fluid and evidence fibrinous stranding on ultrasound. Given re-accumulation, medical pleuroscopy under moderate sedation with TPC placement was performed (Fig. 5).

**Fig. 6:**
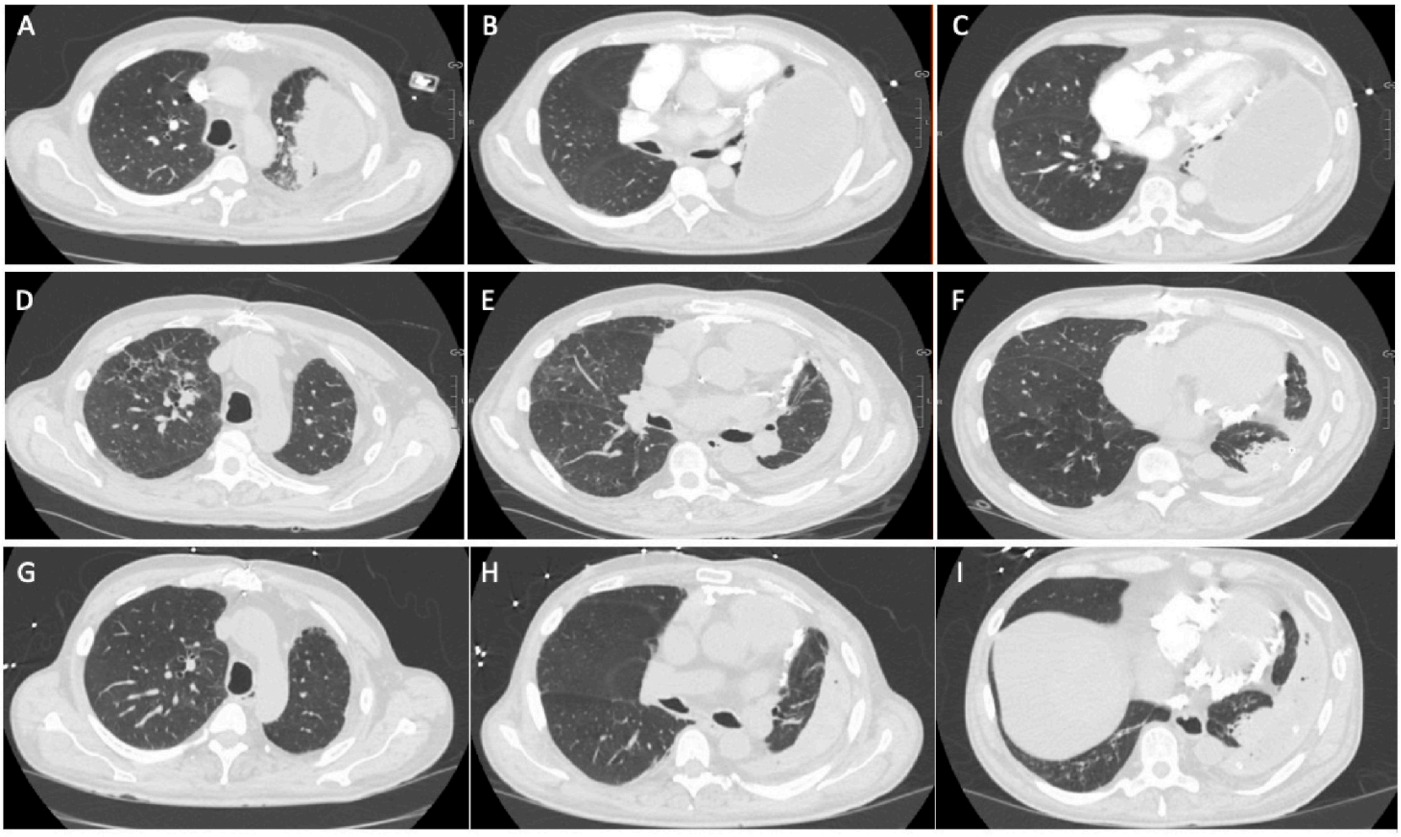
A–C: Chest CT scan on initial referral to our pleural clinic, demonstrating a large loculated effusion. **D-F** Chest CT scan four-months following tunneled pleural catheter placement demonstrating pleural symphysis. **G-I** Most recent chest CT scan available, 17-months following TPC placement, showing pleural symphysis with some associated rounded atelectasis.

Following TPC placement, the patient did well from a respiratory standpoint, with resolution of his dyspnea on exertion, ongoing relief from weekly pleural drainage, and evidence of pleural symphysis on CT scan (Fig. 6).

Following TPC placement he had no further admission for shortness of breath or pleural interventions. His TPC remained in place until his death from hepatocellular carcinoma two years after initial encounter.

## 4 Discussion

Surgical intervention is considered the standard of care for persistent pleural infections [7], however, this is not always feasible in our medically-complex and aging populations. Fortunately, recent advances in local interventions, such as intrapleural lytic therapy [4,9,10], have expanded the therapeutic options for empyema in non-surgical candidates. However, even with lytic therapy, there is a subset of patients with persistent empyema that presents an ongoing management challenge. Additionally, the use of lytic therapy may be limited by other risk factors such as anti-coagulation [11], as seen in our second case. Current AATS guidelines for management of persistent empyema in patients who are not surgical candidates suggest the use of an “empyema tube,” which is done by inserting a chest tube and then cutting the tube near the skin, securing it in place and allowing for open, continuous pleural drainage [7]. This often necessitates continued hospitalization for ongoing management and is not common practice at our institution. Our experience, as outlined in these two cases, has demonstrated the potential role of utilizing tunneled pleural catheters placed during medical pleuroscopy for chronic empyemas when lytic therapy fails and surgery is not an option. Our experience is similar to that reported by Majid et al. who described their single-center retrospective study and noted use of TPC in 11 patients for chronic pleural infection with non-expandable lung [12], though, of these 11 patients, only two had the TPC placed under medical pleuroscopy.

There is limited literature on the use of indwelling pleural catheters in chronically infected spaces, however there has been some discussion regarding infection management of indwelling catheters when TPCs themselves become infected. A modified Delphi consensus statement from CHEST 2020 recommended keeping TPCs in place in the setting of both localized soft-tissue infections and deep pleural space infections. Additionally, this consensus statement recommended that TPC-related pleural space infections be managed first by attempting increasingly aggressive drainage, followed by use of intrapleural fibrinolytic therapy, rather than TPC removal [13]. These recommendations suggest that having a TPC in place does not hinder source control for infected pleural spaces, and, in fact, supports the feasibility of using TPCs in a chronically infected pleural space, as demonstrated in our two cases.

Beyond being safe in pleural infections, TPCs may promote symphysis of the pleural space, which, in turn, can help facilitate source control in empyema. In the IPC-Plus trial, which evaluated patients with malignant pleural effusion, 23% of patients had pleurodesis with TPC alone at 35 days post procedure [14]. This demonstrates that placement of a TPC can result in pleural space obliteration. The AMPLE-2 trial also identified a small subset of patients who achieved symphysis of their pleural space with TPC placement despite trapped lung physiology, lending credence to the notion that a TPC can obliterate the pleural space even in the presence of a trapped lung [15]. This is particularly important given that chronic pleural infection with subsequent pleural thickening can result in trapped lung physiology. Despite this association, the AMPLE-2 trial data suggest that, even in these individuals with trapped lung physiology, there may still be a role for TPC placement.

Beyond source control, TPC placement can provide symptomatic relief for patients with chronic empyema. TPC placement, much like a classic empyema tube or the more morbid surgical Eloesser flap, allows for continuous pleural access for serial sampling and drainage of the pleural space needed for source control. However, unlike an empyema tube, TPCs enable patients to be managed at home and allow for increased mobility and decreased risk of dislodgement, likely positively impacting patient quality of life. Though there is limited literature noting the impact on quality of life in chronic pleural infections, there is ample evidence that use of TPCs in chronic malignant effusions increases patients’ quality of life, decreases hospital stay, and allows for increased mobility and patient independence [16]. This improvement in quality of life was seen in our two case examples.

In our cases we placed a TPC at the time of medical pleuroscopy. We believe the utilization of pleuroscopy with saline washout in conjunction with TPC placement facilitated source control through a minimally invasive approach. Pleural access at the time of TPC placement through the 15.5 Fr peel away trocars, which was the technique in our two cases, has been demonstrated [17] and is similar in incision size to a Seldinger-based pigtail placement, often only requiring moderate sedation. Despite this minimally invasive technique, pleural irrigation and adhesion takedown was still achieved. There is increasing observational data to suggest that medical pleuroscopy is safe and effective in empyema [18,19]. A small randomized control trial by Kheir et al. demonstrated that, when compared to intrapleural lytic therapy, medical pleuroscopy was safe and may shorten hospital stay [20].

In our clinical cases pleuroscopy allowed for saline washouts and take down of simple adhesions, while TPC placement helped with ongoing symptom control and pleural symphysis. We suggest the use of tunneled pleural catheters with placement at time of pleuroscopy may be an effective alternative for management of chronic empyema in patients who are not surgical candidates.

## 5 Conclusion

In review of the literature, there is an increasing body of evidence to suggest that medical pleuroscopy for management of empyema is a safe alternative to VATS in select populations. Our cases, along with the literature available, suggest that the use of tunneled pleural catheters placed at the time of pleuroscopic washout may be an effective means of management for chronic empyema in patients who are not surgical candidates and have not achieved source control with intrapleural lytic therapy. Additional comparative studies evaluating TPC utilization in chronic pleural infections may be warranted.

## Conflicts of interest

The authors have no financial conflicts of interest of funding to disclose.

## Declaration of competing interest

The authors whose names are listed immediately above certify that they have NO affiliations with or involvement in any organization or entity with any financial interest (such as honoraria; educational grants; participation in speakers’ bureaus; membership, employment, consultancies, stock ownership, or other equity interest; and expert testimony or patent-licensing arrangements), or non-financial interest (such as personal or professional relationships, affiliations, knowledge or beliefs) in the subject matter or materials discussed in this manuscript.
